# Impact of comorbidities on risk of angioedema without urticaria in elderly patients

**DOI:** 10.1186/s13223-021-00637-z

**Published:** 2021-12-14

**Authors:** Andrzej Bożek, Magdalena Zając

**Affiliations:** 1 Clinical Department of Internal Disease, Dermatology and Allergology, Medical University of Silesia, Katowice, Poland; 2 Wroclaw Medical University, Wrocław, Poland; 3 MC Sklodowskiej 10, 41-800 Zabrze, Poland

**Keywords:** Angioedema, Elderly, IgE, Hyperuricemia, Hashimoto

## Abstract

**Background:**

Angioedema without urticaria (AWU) is a disease found in the elderly population but is still poorly studied. The aim of this study was to investigate potential factors, especially comorbidities, that may affect the induction of angioedema without urticaria in patients over 60 years of age.

**Methods:**

This was an observational, retrospective study of 242 patients with a diagnosis of AWU and 263 controls. The inclusion criteria were as follows: at least one episode of confirmed AWU based on the ICD-10 code (T78.3) that required treatment in the last 15 years (2004–2019); age above 60 years; detailed medical history of comorbidities; and details regarding the use of drugs at that time. Serum functional and quantitative C1 inhibitor assays were performed, and serum C4 was measured.

Comorbidities were grouped into the following panels: autoimmune, cancer, cardiac, metabolic, respiratory and allergic, liver failure and renal failure. Individual diseases were checked according to ICD code and treatment.

**Results:**

In 1 (0.4%) patient, hereditary angioedema was confirmed. Decreased levels of C1INH were observed in 4 (1.65%) patients, dysfunction of C1INH was observed in 5 (1.76%) patients, and low levels of C4 were observed in 9 (3.71%) patients in the study group. The multiple logistic regression model revealed that patients with hyperuricemia or Hashimoto’s disease had a significantly higher chance of angioedema (OR = 3.21, 95% CI 2.92–3.66, p = 0.002; OR = 1.78 95% CI 1.37–2.21, p = 0.034, respectively).

**Conclusion:**

The obtained results may indicate a significant influence of hyperuricemia or Hashimoto’s disease on angioedema manifestations.

## Background

Angioedema is a common skin disease that is characterized by well-demarcated areas and nonpitting edema of subcutaneous and/or submucosal tissues. The prevalence of angioedema is estimated at 2–20% of the population, which depends on the method of analysis, for example, with or without concomitant urticaria. Angioedema is considered one of the forms of urticaria [1–4].

Angioedema is classified as acquired or hereditary [2, 3]. The first form is allergic associated with anaphylaxis, nonallergic with or without urticaria, drug-induced (especially by angiotensin-converting enzyme inhibitors), complement-mediated secondary to acquired deficiency of C1 inhibitor (C1INH) and idiopathic. Hereditary angioedema (HAE) is caused by C1INH deficiency (decreased or dysfunctional C1INH). However there is also HAE with normal C1-INH with normal levels of C1-INH function and C4. Angioedema is also classified according to mechanism as histamine-mediated and bradykinin-mediated. It should be recognized that the causes of many cases of secondary angioedema are attributable to more than just IgE and its induction by classic allergic mediators [1–3].

The appearance of angioedema in elderly patients can be diverse. It seems that mast cell mediator-mediated angioedema is more common in this age group [2, 3].

Drugs are common triggers for angioedema, and the list of these triggers is long. However, there is not much information on other factors that can influence angioedema in patients over 60 years of age. Hereditary angioedema can still occur late despite a negative family history.^5^

The objective of this study was to investigate potential factors that may affect the induction of angioedema without urticaria (AWU) in patients over 60 years of age. We particularly focused on the possible impact of multimorbidity, which is common in this group of patients, and we excluded those cases that were induced by drugs.

## Methods

### Study design and participants

This was an observational, retrospective study of 242 patients with a diagnosis of AWU. A total of 20,822 patients’ medical charts from geriatric and allergy and primary health outpatient clinic databases were screened according to ICD codes and the inclusion criteria (see below) between 2004 and 2019. The data were fully anonymized before assessment. All patients provided informed written consent to use data from their medical records in the study. The study was approved by the local ethics committees of the Medical University of Silesia in Poland (KNW/0034/2011).

The inclusion criteria were as follows: at least one episode of confirmed AWU based on ICD-10 (T78.3) that required treatment in the last 15 years (2004–2019); age above 60 years; detailed medical history of comorbidities and drugs used at that time; serum functional and quantitative C1INH assay; and serum concentration of C4.

The exclusion criteria were angioedema with chronic urticaria, mild angioedema-like symptoms such as swelling of the tongue or mouth without visible edema, documented angioedema induced by drugs, use of convertase inhibitors or their derivatives, edema reactions that could not be clearly classified as angioedema, deficiencies in documentation, and lack of consent. However, patients whose discontinuation of convertase inhibitors or other suspected drugs did not result in resolution of the edema symptoms were enrolled in the study.

Additionally, hereditary angioedema was also checked based on the ICD-10 code. Clinical diagnosis was based on the following medical history points: (1) a positive family history; (2) onset of symptoms in childhood/adolescence; (3) painful abdominal symptoms; (4) occurrence of upper airway edema; (5) lack of response to antihistamines, glucocorticoids, or epinephrine; (6) presence of prodromal signs or symptoms before swelling; and/or (7) absence of urticaria [2, 3].

The control group was composed of a random sample selected from all patient charts from the primary health outpatient clinic as a part of the same specialist clinics (geriatric and allergy) where patients from the study group were analyzed. Those analyses were performed at same time. The inclusion criteria for the control group were as follows: no primary or secondary diagnosis of angioedema and/or urticaria (including induction by drugs) and age > 60 years old.

The groups were designed in terms of size (according to the formula: n = 16 σ^2^/d^2^) and compliance only in terms of age and sex.

In this way, 423 patients were selected into the prestudy group and 436 into the precontrol subgroup from the initial medical chart analysis. Finally, 242 patients were randomly selected to form the final group for analysis, and 263 similar controls were selected for further analysis by the use of a computer program (Microsoft Allocation Software, Poland).

The detailed characteristics are presented in Table 1.

**Table 1:** Characteristics of the study and control groups

	Study group, n = 242	Control group, n = 263	ASD
Mean age (range; in years)	68.3 (60–88)	65.9 (60–91)	0.120
Mean BMI (kg/m2) ± SD	24.1 ± 6.4	27.2 ± 5.8	0.151
Female (%)	138 (57)	152 (58)	0.006
Rural area (%)	115 (48)	140 (53)	0.081
Smoker or former smoker (%)	97 (40)	99 (37)	0.012
Comorbidities (%)			
One chronic disease	65 (27)	74 (28)	0.181
Two chronic diseases	110 (45)	121 (46)	0.176
> Two chronic diseases	67 (28)	83 (32)*	0.048
No. of angioedema incidences (%)		–	–
One	54 (22)		
Two to five	161 (67)		
Six or more (recurrent)	27 (11)		
Emergency intervention	48 (20)	–	–
Hospitalization due to angioedema	63 (26)		
Mean time of onset in minutes (range)	18 min (2–129)		
Localization			
Mouth	179 (74)		
Eye area	104 (43)		
Throat	62 (26)		
Hands or feet	75 (31)		
Respiratory tract	32 (13)		
Digestive tract	29 (12)		
Others	19 (8)		
Symptomatic medications used to treat angioedema (%)		–	–
Antihistamines	219 (90)		
Adrenaline	17 (7)		
Systemic steroids	114 (47)		
Calcium	176 (73)		
Montelukast	51 (21)		
Tranexamic acid	11 (5)		
Blood plasma	2 (1)		

*ASD *absolute standardized difference, *SD* standard deviation, *BMI* body mass index

*Borderline statistical significance

### Variables analyzed

Individual disease entities were grouped into the following panels: autoimmune, cancer, cardiac, metabolic, respiratory and allergic, liver failure and renal failure.

The autoimmune panel contained systemic lupus erythematosus, diabetes type 1, rheumatic arthritis, psoriasis/psoriatic arthritis, multiple sclerosis, inflammatory bowel diseases (Crohn’s disease, ulcerative colitis), Addison’s disease, Graves’s disease, Sjogren’s disease, Hashimoto’s disease, autoimmune vasculitis, polymyositis, celiac disease, pernicious anemia and myasthenia gravis.

The cancerous panel contained lung cancer, nonmelanoma skin cancers, melanoma, breast cancer, leukemia (chronic myeloid leukemia, chronic lymphocytic leukemia, lymphoma), breast cancer, prostate cancer, and colon cancer.

Cardiovascular panel: arterial hypertension, heart failure, coronary disease.

Metabolic panel: hyperuricemia, type 2 diabetes, hyperlipidemia, overweight (BMI > 25).

Respiratory and allergic panel: chronic obstructive pulmonary disease (COPD), bronchial asthma, emphysema, allergic rhinitis, atopic dermatitis, eczema.

Liver failure panel: cirrhosis, viral hepatitis type B or C, toxic liver failure.

Renal failure panel: lupus nephropathy, polycystic kidney disease, nephrotic syndrome, chronic urinary tract problems.

To avoid bias, a disease was counted as present if two criteria were met: diagnosis with the corresponding ICD-10 code and appropriate treatment undertaken.

For this same reason, unlisted diseases that occurred in fewer than 1% of patients in both populations and were insignificant as a disease phenomenon in both populations due to the small number of patients were excluded from the analysis.

Additionally, retrospective results of the C4 and quantitative and functional C1INH levels in blood serum were also analyzed.

### Statistical analysis

The data were analyzed with a statistical software package (STATISTICA version 8.2, StatSoft, Cracow, Poland). The study group and control group were compared by using absolute standardized differences (ASDs) in age and sex. If the ASD was < 0.1, the characteristics of both groups were considered to be similar.

To explore the association between the presence of angioedema and the analyzed concomitant diseases using the C1INH and C4 results, a multiple logistic regression model was estimated, and the results are presented as odds ratios (ORs) and 95% confidence intervals (95% CIs). First, a univariate analysis was carried out to see how each potential variable (age range, sex, BMI, disease groups as the panels described above, as well as particular diagnosed diseases within them, low C1INH, dysfunction of C1INH and low C4) affected the probability of AWU.

All these variables were categorical except age, C1INH, and C4. The association between outcome and explanatory variables was analyzed with the χ2 test, and odds ratios and corresponding 95% confidence intervals were calculated. Logistic regression analysis was used to adjust the effect of intercorrelation between variables on isolated angioedema. Variables that were not significant in this analysis were excluded. In the second step, variables found to be statistically significant (presented in Table 2) were tested in a combined analysis to determine whether they had a weak or strong influence on angioedema.

**Table 2:** Multiple logistic regression for the risk of angioedema without urticaria in the analyzed elderly patients

Variables significant after univariate analysis	Risk of angioedema without urticaria	
	OR (95% CI)	p
BMI	0.96 (0.75–1.19)	0.157
Comorbidities with more than two chronic diseases	1.18 (0.82–1.34)	0.056
Autoimmune panel	1.27 (0.79–1.59)	0.081
Metabolic panel	1.56 (1.18–1.59)	0.073
Inflammatory bowel disease	1.21 (1.10–1.52)	0.062
Hashimoto’s disease	1.78 (1.37–2.21)	0.034
Autoimmune vasculitis	1.11 (0.89–1.61)	0.069
Leukemia*	0.88 (0.53–1.36)	0.042
Hyperuricemia	3.21 (2.92–3.66)	0.002
Breast cancer	0.96 (0.41–1.29)	0.110
Atopic dermatitis	0.98 (0.76–1.55)	0.107

^*^The term “leukemia” included chronic myeloid leukemia, chronic lymphocytic leukemia, and lymphoma

## Results

A total of 242 patients with AWU and 263 controls were finally analyzed. Table 1 shows the demographic characteristics of the patients. There were no statistically significant differences between the study and control groups (p < 0.05).

In 1 (0.4%) patient, hereditary angioedema was confirmed. Importantly, low levels of C1INH were observed in 4 (1.65%) patients, dysfunction of C1INH was observed in 5 (1.76%) patients, and low levels of C4 were observed in 9 (3.71%) patients in the study group. However, in 2 (0.7%) patients in the control group, low levels of C4 in serum were also noticed. However, in all these cases, the reductions in C1INH or C4 values were slightly below the laboratory norm. Hereditary angioedema was rechecked and excluded according to the criteria described in the methods.

In the univariate analysis, the following variables affected the probability of AWU: BMI (odds ratio (OR) = 1.41, 95% confidence interval (CI) 1.28–1.61, p = 0.022), more than two comorbid chronic diseases (OR = 1.61, 95% CI 1.38–1.79, p = 0.02), the autoimmune panel (OR = 2.22, 95% CI 2.11–3.28, p = 0.041), the metabolic panel (OR = 3.10, 95% CI 2.62–3.52, p = 0.029) and separately diagnosed diseases mentioned in those panels: inflammatory bowel diseases (OR = 1.76, 95% CI 1.28–2.15), Hashimoto’s disease (OR = 2.19, 95% CI 184–2.29, p = 0.031), autoimmune vasculitis (OR = 1.42, 95% CI 1.22–1.78, p = 0.043), leukemia (OR = 2.62, 95% CI 1.78–2.98, p = 0.012), breast cancer (OR = 1.21, 95% CI 1.56–1.93, p = 0.046), hyperuricemia (OR = 3.92, 95% CI 3.70–428, p = 0.002) and atopic dermatitis (OR = 1.38, 95% CI 1.19–1.62, p = 0.035).

The remaining disease panels and individual diseases (as described in the methods) did not significantly influence the risk of angioedema without urticaria, and these data were excluded from further analysis (for example, the cardiovascular panel).

The multiple logistic regression model revealed that patients with hyperuricemia or Hashimoto’s disease had a significantly higher chance (OR = 3.21, OR = 1.78) of angioedema (see Table 2). There were 57 (24%) patients with hyperuricemia, 43 (17%) with Hashimoto’s disease and 21 (9%) with both diseases. In controls, these respective numbers were as follows: 34 (13%), 27 (10%) and 8 (3%).

## Discussion

The search for interrelationships between different diseases is difficult, especially in elderly patients. Comorbidities, polypharmacy, and the lack of focus on less severe diseases by patients and doctors may make it difficult to accurately assess such relationships. Similar difficulties arise in the assessment of how various factors influence the manifestation of AWU in patients over 60 years of age. For this reason, many patients with suspected or proven drug-related edema were excluded from the study.

The obtained results confirmed a moderate relationship between angioedema and the examined diseases and other analyzed features. This is in line with the current literature [1, 4]. When performing the initial overall analysis, the dominant group of patients was patients with drug-induced angioedema, and only 1 patient had hereditary angioedema.

Multiple logistic regression revealed a significant odds ratio for angioedema in elderly subjects with hyperuricemia. This is probably the first such observation.

Many studies have focused on the association between hyperuricemia and the components of metabolic syndrome, including diabetes, overweight, arterial hypertension, nephropathy and cardiovascular diseases. Hyperuricemia, usually defined as a uric acid concentration exceeding 7.0 mg/dl, is often also observed in patients over 60 years of age [6, 7].

The mechanisms of the harmful effects of uric acid on the blood vessel wall include damage to the endothelium leading to endothelial production of nitric oxide (NO), activation of thrombocytes and increases in hydrostatic pressure. In addition, uric acid is proinflammatory: it stimulates the synthesis of cytokines such as chemotactic factor monocytes 1 (MCP-1, monocyte chemotactic protein 1), interleukin-1b (IL-1b), IL-6 and tumor necrosis factor alpha (TNF-α), which may influence the initiation of endothelial inflammatory processes [8, 9]. Hypothetically, these mechanisms may occur in small vessels, e.g., in the skin, increase vascular permeability and induce an inflammatory process that may lead to regional angioedema. In these patients, there was no secondary decrease in the concentrations of C1INH and C4 (data not presented in detail). Testing this hypothesis requires an appropriate prospective study that is currently underway. In the literature, there is no direct information about the role of metabolic syndrome and its influence on the manifestations of angioedema. However, only the influence of glycemic disturbances on the appearance of angioedema was confirmed [10]. In the present study, the presence of diabetes did not significantly increase the risk of angioedema manifestation, but serum glucose levels were not analyzed.

Hashimoto’s disease also contributed significantly to the greater appearance of angioedema but to a lesser extent than hyperuricemia. This phenomenon has been observed previously [3, 11, 12]. Authors have suggested that thyroid cells have Toll-like receptors that respond to pathogen- and damage-associated molecular patterns. These triggers activate innate immunity, CD4 + lymphocytes, and the production of IL-1β, IFN-γ, and TNF-α, which subsequently induce cell damage and apoptosis, which are key mechanisms for the development of angioedema and/or urticaria [11–13]. The coexistence of angioedema and autoimmune diseases may have a similar mechanism as that in patients with chronic spontaneous urticaria (CSU) described by Bracke et al. [14]. The presented concept of “overlapping autoimmune diseases” suggests that disorders that are autoimmune in nature, for example, urticaria (or angioedema), occur at increased frequency in patients with known autoimmune diseases. The presence of autoantibodies on a background of chronic inflammation (for example, anti-TPO) is a potential mechanism. Autoallergy is also possible in patients with CSU and Hashimoto’s disease because these patients demonstrated higher levels of IgE anti-thyroid peroxidase antibodies in comparison to controls. Unfortunately, this mechanism was not investigable in this study.

The final analysis shows a small group of patients with complement-mediated angioedema secondary to acquired deficiency of C1INH, a relatively new finding. However, in this retrospective analysis, we only had single results for the C1INH and C4 determinations, which did not cover any of their changes. Furthermore, there was only one person with hereditary angioedema, which is consistent with the prevalence of this condition in the general population [15].

There were some limitations to this study. As described above, despite strict adherence to the inclusion criteria, it was impossible to exclude single cases where angioedema has been associated with the use of drugs, especially those that had been used occasionally but not included in the history of the disease. Additionally, the impact of other diseases on angioedema that have not been taken into account due to their rarity was also possible. Only C1INH and C4 in blood serum were directly analyzed in the study, while other biochemical parameters, such as IgE, TSH, glucose, and uric acid, were only used to diagnose the disease, but the values themselves were not statistically analyzed in this study.

The obtained results may indicate a significant influence of hyperuricemia and Hashimoto’s disease on the manifestations of angioedema. Uric acid is pro-inflammatory and vascular damaging, and the presence of anti-thyroid antibodies may induce autoallergy. In this group of elderly patients with AWU, a small number of patients with decreased levels of C1INH and C4 were notable. Further prospective studies are needed to confirm these observations.

## Data Availability

All data used to support the findings of this study, including potentially identifying and sensitive patient information in analyzed records, may be released upon request from the insurer Pro Ins in Poland despite the consent of the Bioethical Committee Katowice, Medical University of Silesia Poland. Queries should be directed to: Email: p.o.j.a.n.ins@wp.pl.

## Abbreviations

AWU: Angioedema without urticaria
C1INH: C1 inhibitor
CSU: Chronic spontaneous urticaria
OR: Odds ratio
MCP1: Chemotactic factor monocytes
NO: Nitric oxide
TNF α: Tumor necrosis factor alpha
